# MowJoe: a method for automated-high throughput dissected leaf phenotyping

**DOI:** 10.1186/s13007-018-0290-y

**Published:** 2018-03-26

**Authors:** Henrik Failmezger, Janne Lempe, Nasim Khadem, Maria Cartolano, Miltos Tsiantis, Achim Tresch

**Affiliations:** 1 Max Planck Institute for Plant Breeding Research, Carl-von-Linné-Weg 10, 50829 Cologne, Germany; 2 Department of Biology, University of Cologne, Zülpicher Str. 47, 50674 Cologne, Germany; 3 Institute of Medical Statistics and Computational Biology, University of Cologne, Bachemer Strasse 86, 50931 Cologne, Germany

## Abstract

**Background:**

Accurate and automated phenotyping of leaf images is necessary for high throughput studies of leaf form like genome-wide association analysis and other forms of quantitative trait locus mapping. Dissected leaves (also referred to as compound) that are subdivided into individual units are an attractive system to study diversification of form. However, there are only few software tools for their automated analysis. Thus, high-throughput image processing algorithms are needed that can partition these leaves in their phenotypically relevant units and calculate morphological features based on these units.

**Results:**

We have developed MowJoe, an image processing algorithm that dissects a dissected leaf into leaflets, petiolule, rachis and petioles. It employs image skeletonization to convert leaves into graphs, and thereafter applies algorithms operating on graph structures. This partitioning of a leaf allows the derivation of morphological features such as leaf size, or eccentricity of leaflets. Furthermore, MowJoe automatically places landmarks onto the terminal leaflet that can be used for further leaf shape analysis. It generates specific output files that can directly be imported into downstream shape analysis tools. We applied the algorithm to two accessions of *Cardamine hirsuta* and show that our features are able to robustly discriminate between these accessions.

**Conclusion:**

MowJoe is a tool for the semi-automated, quantitative high throughput shape analysis of dissected leaf images. It provides the statistical power for the detection of the genetic basis of quantitative morphological variations.

**Electronic supplementary material:**

The online version of this article (10.1186/s13007-018-0290-y) contains supplementary material, which is available to authorized users.

## Background

Plant leaves are critical for survival as they are the primary site of photosynthesis. Leaf shape and size show tremendous variation between species, which is assumed to be the result of adaptive evolutionary processes tinkering with leaf shape to allow best performance in particular ecological niches [12, 14, 20, 23, 36]. Therefore, plant leaves have attracted scientists from diverse disciplines to study ecology, evolution, development and patterning mechanisms [5, 17, 21, 22, 27, 34, 40, 41].

Qualitative descriptions of leaf shape, traditionally used for species classification, are insufficient to characterize the developmental and genetic factors underlying phenotypic variation. Rather, it is necessary to quantitatively describe the geometric features of leaf shape and perform shape analysis [6, 26]. Easily accessible measures include length, width, perimeter and area. A more refined morphometric analysis, however, is based on the extraction of multivariate shape features. Typically these methods analyse the relative position of landmarks—homologous points identified in each leaf sample—or sequential positions along the leaf outlines or combinations of the two. These approaches are collectively referred to as geometric morphometrics. Examples of methods based on outline analysis are Eigenshape analyses and elliptic Fourier analysis [19, 24, 31].

**Fig. 1:**
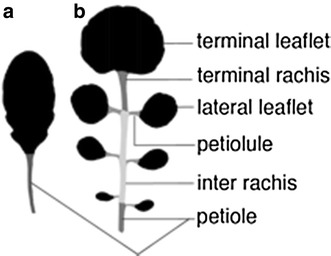
Leaf architecture of simple leaves (**a**) and dissected leaves (**b**) of *A. thaliana* and *C. hirsuta* respectively. The simple leaf consists of a single entire leaf blade and a petiole. The architecture of a dissected leaf is more complex. The leaf blade is divided into terminal and several lateral leaflets, which are arranged along the main axis, which itself consists of the terminal rachis, inter rachis and the petiole. The petiolules connect the rachis and the lateral leaflets

**Fig. 2:**
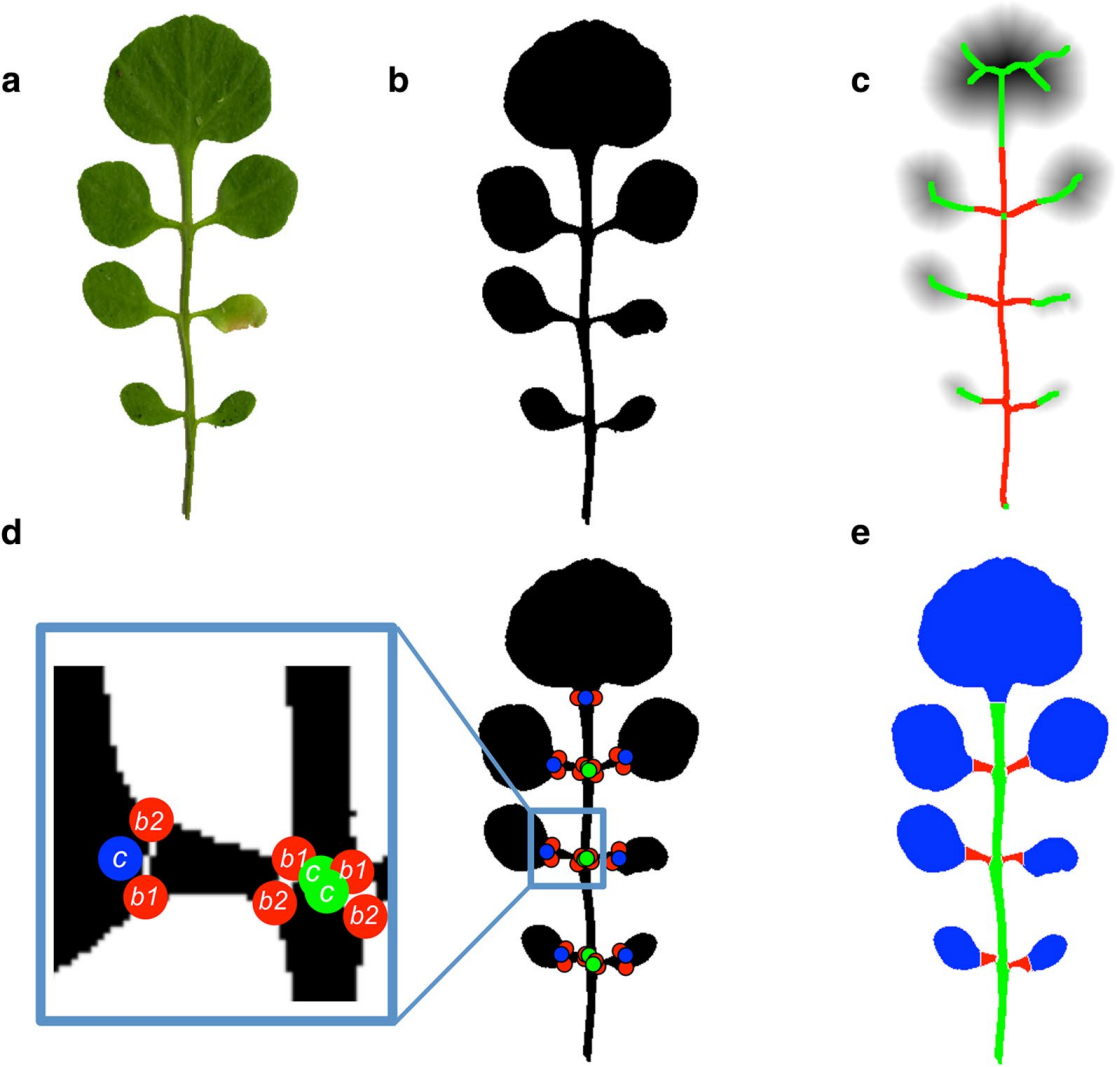
Leaflet segmentation. The scanned leaf (**a**) is scaled and converted to a binary image (**b**). The foreground pixels of the binary image are distance transformed (**c**). The darker the grey scale of a point, the larger its distance to the outline of the leaf. Skeleton pixels are classified according to their distance transform value (red = small, green = large). **d** Cut nodes (blue, *c*) are points on the skeleton that mark the border between leaflet pixels and petiolule pixels. Branching nodes (green, *c*) are points on the skeleton that mark the border between petiolule pixels and rachis pixels. **e** The pairs of red points (*b*1, *b*2) cut the leaf into leaflets (blue areas), petiolules (red areas) and rachis (green)

For the identification of loci controlling quantitative traits, phenotypic analysis of larger populations with proper randomization is necessary, for example in quantitative trait locus (QTL) mapping or genome-wide association (GWA) studies. Sizes of mapping populations and availability of genetic information on such largely increased with the advancement of sequencing technologies and phenotyping has become the bottleneck. Whereas simple phenotypes can be determined by eye, more complex quantitative phenotypes need to be defined algorithmically. During the last years, several tools were developed which measure, e.g., leaf size [16, 29], or more complex shape parameters, and shape landmarks [2, 8, 9, 18, 40, 42]. Most of these tools calculate features for simple leaves, in which the leaf blade is entire (Fig. 1a), and they are well established for plants like *Arabidopsis thaliana*. However, dissected leaves, leaves that are divided into several units so called leaflets (Figs. 1b, 2a), move more and more into focus [3, 10, 22], as they allow the investigation of interesting questions on shape evolution, leaf patterning and development.

In order to perform quantitative shape analyses of dissected leaves in larger populations, we have developed the MowJoe analysis pipeline. As compared to entire leaves, the shape of dissected leaves is more challenging to image processing algorithms. Instead of reporting merely morphological features of the entire leaf, one needs to cut the dissected leaf into pre-defined, phenotypically meaningful parts beforehand. A recent approach [11] relies on the assumptions of circular leaflets and the symmetric positioning of leaflets along the rachis to build a leaf shape model. Leaflets are then segmented by Active Contours. Another approach searches for concavity points in the leaf and partitions the leaf based on these points [35] or deletes the rachis of the leaves by fitting a polynomial curve [1]. The drawback of these methods is their sensitivity to violations of leaflet convexity. These may occur naturally or through imaging artifacts like fissures in the leaf produced by its fixation to a plain surface.

Recently, skeletonization of an image was combined with morphological operations in order to measure the length of the branches in rice panicles [13]. Informally, the skeleton is a reduced, one-dimensional representation of a leaf through its “central” points (see Fig. 2c). We use skeletonization to derive marker points on the skeleton of a leaf, which mark, e.g., its leaflets. In contrast to [13], we exploit the fact that skeleton points located within a leaflet have a larger distance to the outline of the leaf than skeleton points lying in the rachis or the petiolule. This allows the fast and reliable determination of the points on the skeleton which separate leaflets from petiolules and rachis. Based on this algorithm, we have developed MowJoe, a software tool that segments dissected leaves into phenotypically meaningful units and calculates morphological features for the whole leaf as well as for the individual leaflets, the petiolule, the inter-rachis, the petiole and the terminal rachis. Additionally, it determines landmarks and outlines of the terminal leaflet that can directly be used as input files for downstream shape analysis software, such as MorphoJ [25], Eigenshape Analysis [32] and R shapes [15]. We apply MowJoe to two different accessions of the model plant species *Cardamine hirsuta* [22] and demonstrate the potential of MowJoe to identify leaf shape variation in dissected leaves.

## Results

### Image segmentation

#### Image acquisition and binarization

We obtained digitized 2D color images by scanning leaves of the fifth node. In order to improve the processing time, the images were rescaled by a factor of 25% using a bicubic interpolation (Fig. 2a).

Foreground pixels were extracted by using a method similar to [28]. The image was converted from RGB to HSV space and a 2-means clustering of the pixels in the saturation-value (SV) space was performed. This initial clustering gave an estimate of the intensity centroids of foreground and background pixels in SV space, which served as an initialization of a 2-multivariate Gaussian mixture model on the SV values of the image pixels. The covariance matrices of that model were initialized as scaled identity matrices, the scale chosen as the standard deviation of the whole data set. From the resulting segmentation, the largest connected foreground (green) component was kept (Fig. 2b). The Gaussian mixture method outperformed other common methods such as Otsu thresholding [37] applied to the grey scale or the green component of an image (Additional file 1: Fig. S1).

#### Graph representation of a leaf

The binarized leaf is converted into a skeleton representation. Skeletonization of a binary image is a standard procedure in image processing [39]. Informally, it calculates one-dimensional summary of a 2-dimensional binary image (Fig. 2c). Technically, a distance transform is applied to the image first. This operation assigns to every leaf pixel x the Euclidean distance d(x) to the nearest background pixel. The distance value d(x) can be thought of as the radius of the largest circle centered at x which touches the border of the leaf. Those points whose circle touches the border at least twice are the skeleton pixels (Fig. 2c).

Afterwards, the skeleton is pruned further by removing small branches whose length falls below a threshold of 25 pixels (corresponding to 4.5 mm. This value can be adjusted arbitrarily in the MowJoe GUI). The skeleton is then converted into a graph with one node for every skeleton pixel and one edge for every pair of neighboring pixels in the skeleton. This graph representation of the skeleton simplifies further processing.

Our next task was to segment the skeleton into rachis, petiolule and leaflet parts. To this end, we define a cut node as skeleton pixels in the petiolule neighboring to a leaflet pixel (blue points in Fig. 2d). Similarly, branching nodes are defined as skeleton pixels on the rachis neighboring to a petiolule pixel (green points in Fig. 2d). Skeleton pixels in the leaflet are likely to have larger distance transform values than skeleton pixels in petiolules or in the rachis. This property is exploited to find the cut nodes. The distance values of the skeleton pixels are clustered by 2-Gaussian mixture clustering. This gives a rough segmentation in leaflet pixels *L* (green points in Fig. 2c, having larger distance values) and petiolule/rachis pixels *R* (red points in Fig. 2c, having smaller distance values). Since this separation is not perfect and wrong labeling happens at leaflet nodes that are far from the rachis, we implemented a procedure that removes these errors and gives an optimal separation between leaflet and rachis/petiolule pixels. Note that the labeling of points (leaflet versus petiolule/rachis) should only switch once on a simple path from the rachis bottom towards extremal points in the leaflet (we call nodes in the graph that have only one neighbor extremal; unfortunately, the more common term “leaf node” has different meanings in biology and mathematics). We calculate all paths in the graph starting at the bottom node (end of the rachis) to the extremal leaf nodes. Given such a path, we enumerate the nodes on that path from 1 to N, starting with the bottom node. If node k was a cut point, then all points $1,\ldots ,k$ should be petiolule/rachis points, and all other points should be leaflet points. The number of *M*(*k*) misclassifications according to our initial 2-Gaussian mixture clustering is then

$$\begin{aligned} M(k) = |\left\{ 1,\ldots k\right\} \cap L|+|\left\{ k+1,\ldots ,N\right\} \cap R|. \end{aligned}$$

This yields an easy criterion to robustly identify the correct cut point: it is the (first) node *k* which minimizes *M*(*k*). There might be several paths ending in the same leaflet (e.g., see the terminal leaflet in Fig. 2c), possibly leading to different cut nodes for the same leaflet. However in practice, these cut points always agreed. In case of disagreement, we suggest to use the cut point with the smallest value.

#### Dissection of leaf components

The cut nodes serve for the identification of individual leaflets in the binarized image. To define the boundary of a leaflet, we choose *b*1, the point on the image boundary at minimum distance to a given cut node *c*. Next, we define *b*2 as the boundary point at minimum distance to *c* with respect to the constraint $d(b1,b2)>d(c,b2)$, i.e., *b*2 lies on the “opposite side” of *b*1 (see Fig. 2d). The line between *b*1 and *b*2 separates one leaflet from the rest of the leaf. This finally results in a separation of the leaf into petiole, rachis, petiolule and leaflets (see Fig. 2e). In order to verify the accuracy of our method, we manually measured the rachis and the petiolule of 5 leaves (Additional file 1: Fig. S2) and compared the results to MowJoe’s results. The mean petiolule length was 26.3 pixels (4.6 mm) as measured manually. The mean deviation between MowJow and manual measurements of petiolule length was 2.9 pixels (0.5 mm), corresponding to a mean relative deviation of 11%. For the rachis, the mean length was 368.4 pixels (64.8 mm), and the mean deviation was 2.6 pixels (0.46 mm), corresponding to a mean relative deviation of 0.7%. All in all, manual and MowJoe measurements were in good agreement and did not show any systematic differences.

## Feature extraction

**Fig. 3:**
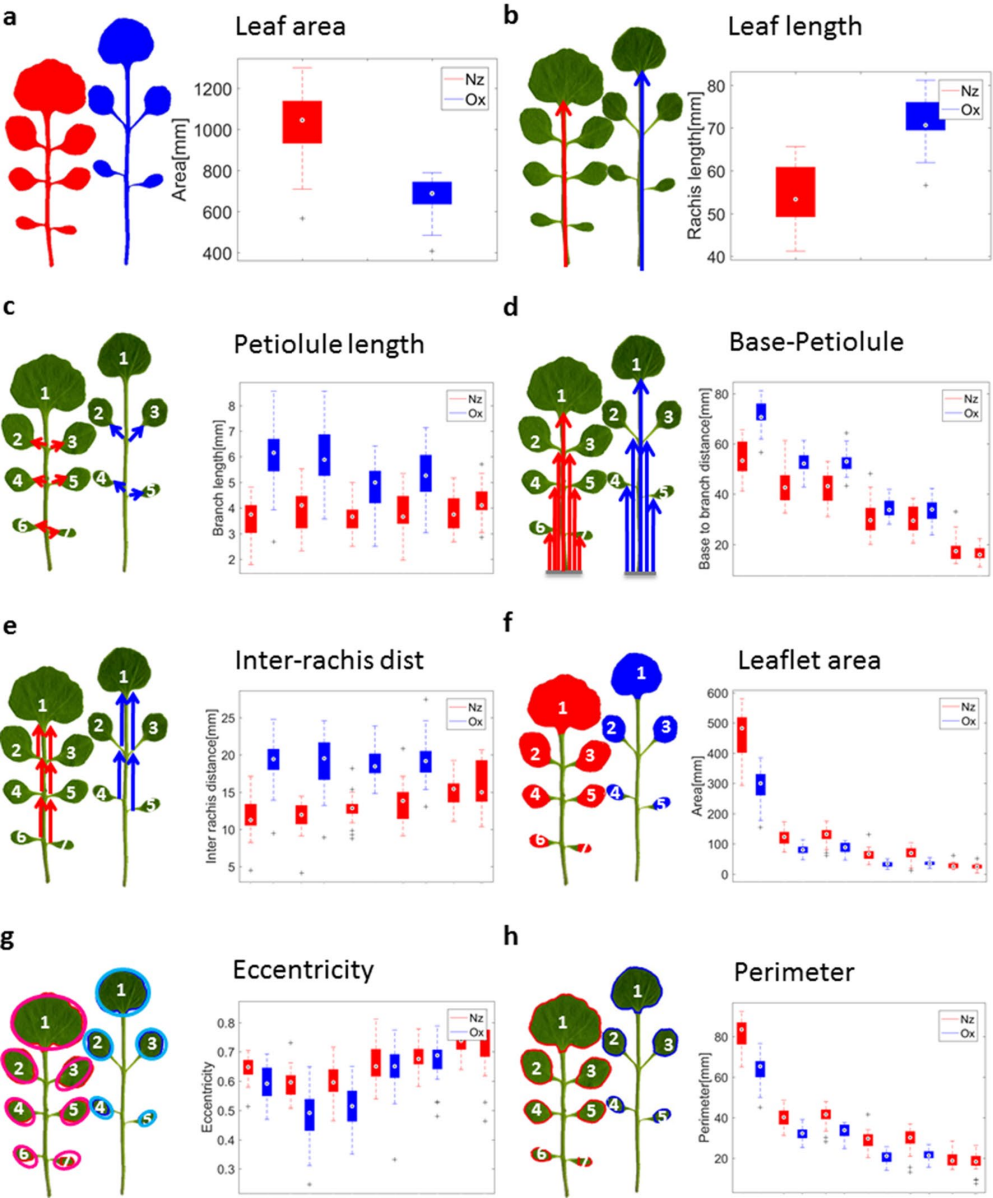
Comparison of accessions by different statistics. Left leaf: Example of the New Zealand (Nz, red) accession, right leaf: Example of the Oxford (Ox, blue) accession. Leaflets are numbered according to their distance to the terminal leaflet. **a** Whole leaf area, **b** length of the rachis, **c** petiolule length, **d** number of pixels from leaf base to branching node, **e** distance from leaflet branching node to next branching node, **f** leaflet area, **g** leaflet eccentricity, **h** leaflet perimeter

The segmentation of the leaf allows the calculation of global phenotypic features of the whole leaf as well as local features of the rachis, petiolules and leaflets. Such features can potentially discriminate different accessions of *C. hirsuta*. We compared global and local features of two accessions of *C. hirsuta* originating from New Zealand (Nz) and Oxford (Ox). For these accessions a global leaf feature like leaf area already provides a good separation (Fig. 3a; Additional file 1: Fig. S3A). While the Nz accession has a larger whole leaf area, Ox leaves have a longer rachis (Fig. 3b). Further, Ox leaves tend to have at most four leaflets whereas Nz have up to seven leaflets. However, local features offer higher discriminatory power and thus boost the discovery of morphologically relevant genes in genome-wide association studies. In order to facilitate the comparison of local features, leaflets were numbered according to their distance to the terminal leaflet and their position (left or right) relative to the rachis. The petiolule length, the number of pixels from leaflet branching node to leaflet cut node, discriminates unequivocally between the leaflets of the Nz and those of the Ox accession (Fig. 3c). Another local feature is the inter-rachis distance, the distance between two neighboring leaflet crossing points (Fig. 3e). Similar to the petiolule lengths, leaves from the Ox accession tend to have larger inter-rachis distances (Fig. 3e). This is also true for a related measure, the distance from the leaflet base point to the leaflet branching points (Fig. 3d). Other interesting features are leaflet shape parameters derived from individual leaflets (Fig. 3f–h). We calculated several morphometric features that describe the shape of a leaflet: Leaflet area (number of pixels) and leaflet perimeter, the length of the leaflet major and minor axis, and leaflet eccentricity. Here, leaflet eccentricity models the pixels of a leaflet by an ellipse of similar shape and same size (formally, the ellipse is obtained from the empirical covariance matrix generated by the coordinates of the foreground pixels). The deviation from a circular shape is measured by the eccentricity, $e=\sqrt{1-\frac{b^{2}}{a^{2}}}$, where a (resp. b) is the length of the major (resp. minor) axis of the ellipse. Since an ellipse captures the essential shape of a leaflet quite well, eccentricity turns out to be a useful descriptor. Leaflets from the Nz accession have larger leaflet areas, major/minor axis lengths, equivalence diameters, perimeters and leaflet eccentricities (Fig. 3f–h; Additional file 1: Fig. S3B,C,D). In summary, we have demonstrated that morphological features, either for the leaflets or the whole leaf, are able to discriminate between the two *C. hirsuta* accessions.

## Morphometric shape analysis

Morphometric shape analysis allows a quantitative description of shape additional to one dimensional size measures. This multivariate analysis makes use of biological homologous points so called landmarks that determine shape. In order to calculate homologous landmarks for each leaf in the MowJoe software, we first searched the top point for every leaf. To do so, we calculated the line from the leaf bottom node to the terminal leaf cut node. The intersection of this line with the terminal leaf outline determined the top point.

**Fig. 4:**
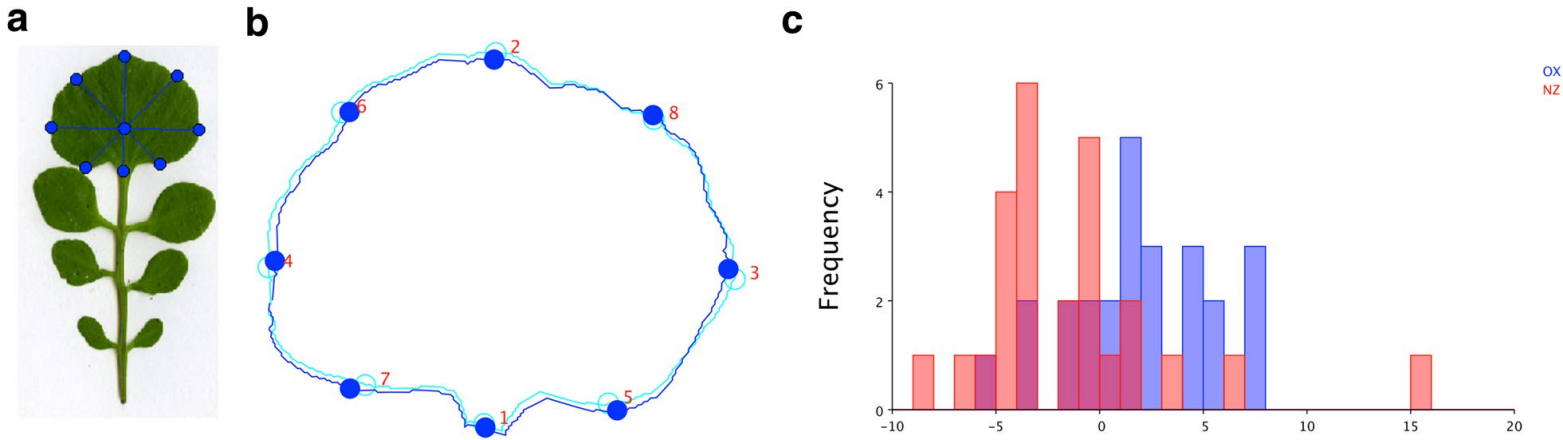
Landmark generation and shape analysis by MorphoJ. 8 Landmarks are generated for the terminal leaflet (**a**). Further shape analysis can be applied by MorphoJ. Alignment of the landmarks for the Nz accession (**b**). Histogram of the discriminant scores of the Fisher’s discriminant function analysis (DFA) for the two accessions (**c**)

Two more landmarks were defined by searching for the longest perpendicular line, and by using the intersection point of this line with the terminal leaflet outline. The lines that formed an angle of 45° to these lines determined the rest of the landmarks (Fig. 4a). The landmarks of the terminal leaflets of all leaves that are exported by MowJoe were further analyzed by MorphoJ [25] (Fig. 4b). MorphoJ first performs a Procrustes fit which removes variation in position, orientation and size from the data. We tested the difference in mean shape between these two accessions in MorphoJ using Fisher’s discriminant function analysis (DFA). We observed a significant difference for the terminal leaflet shape (Fig. 4c, P value: 0.0003).

**Fig. 5:**
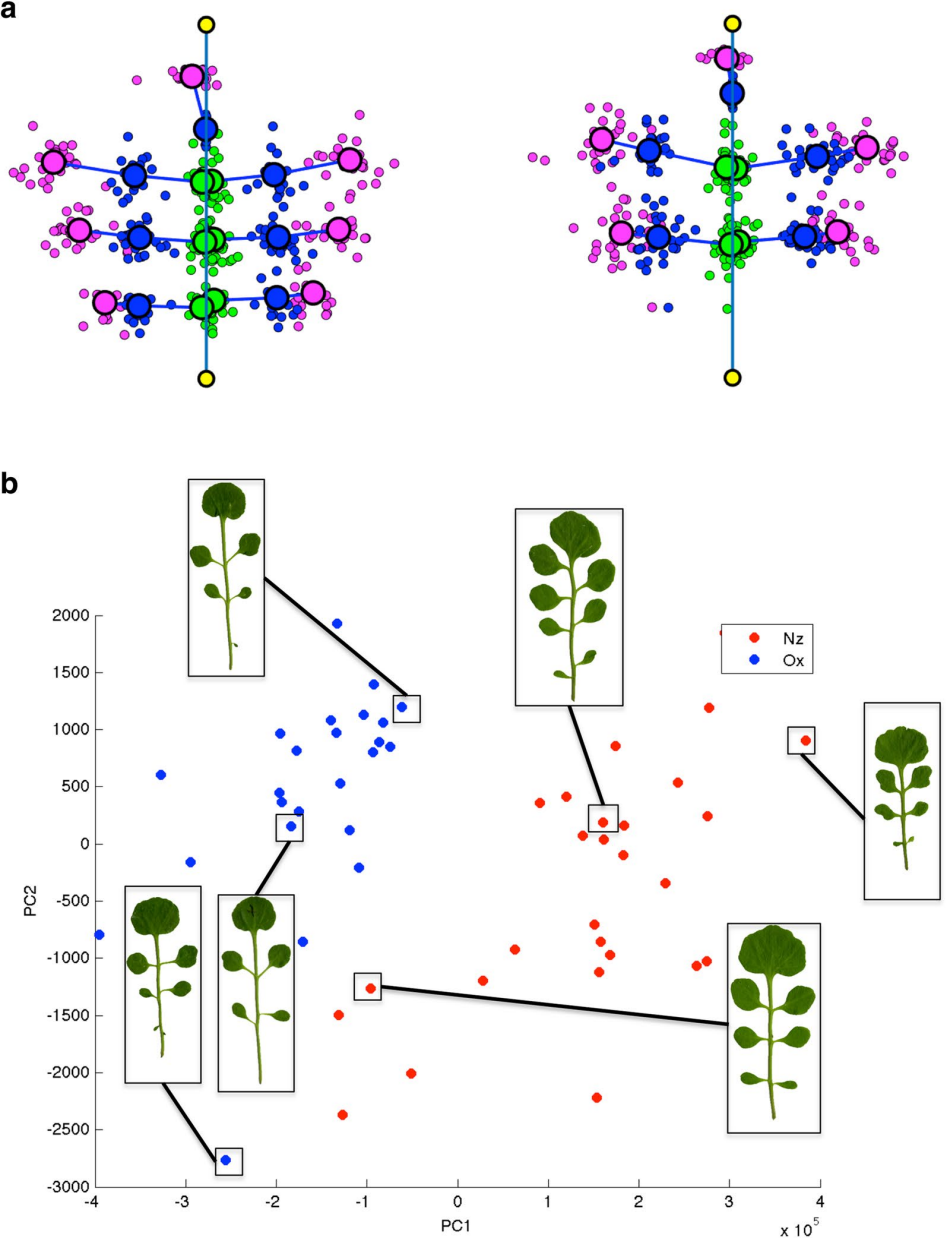
Comparison of combined leaf features. **a** Metaleaf construction for the Nz accession (left) and the Ox accession (right). All marker point positions (magenta: Leaflet centers, blue: Cut nodes, green: Branching nodes, yellow: base/top point of leaf. The bordered points are the respective means of the surrounding points of the same color) of one accession were combined in the same coordinate system, **b** principal component analysis of global leaf features and features of the first three leaflets. Leaflets of the Nz accession are shown in red, leaflets from the Ox accession are shown in blue

For additional shape analysis, MowJoe generates output files that can be imported to Eigenshape analysis [32], or shape analysis implemented in the R package ‘shapes’ [15].

## Comparison of combined leaf features

We generated a unified leaf representation for each accession, by an affine mapping of all the leaf’s marker points into one coordinate system. This transformation is defined by mapping the leaf base point (lower rachis end) to the origin of the 2d plane, and mapping the top point of the leaf to (0,1) on the y-axis. The overlay of all leaf images of one accession creates the so-called “metaleaf”, which provides an overview of the morphological variety of that accession (Fig. 5a). On the metaleaf, the difference between the accessions becomes obvious (Fig. 5a). The Ox accession has larger distances between branching nodes and cut nodes but smaller distances between cut nodes and leaflet centers.

In order to define a low-dimensional Euclidean space that satisfactorily captures the morphological variety of entire leaves, we merged whole leaf features, like leaf area and leaf perimeter, with features derived from individual leaflets. We only included features of the first three leaflets, as the leaves have different numbers of leaflets and we wanted to keep the feature sets comparable. We then performed principal components analysis for further dimensionality reduction. The leaves of the two *C. hirsuta* accessions are clearly separated in this morphological leaf space, indicating that our features extract relevant information (Fig. 5b).

## Discussion

Several tools for leaf size and shape measurements have been developed [2, 4, 7, 33, 42]. However, dissected leaves are more complex, they consist of several distinct morphological units that need to be identified accurately. We developed an image processing algorithm that is able to extract these units from the entire leaf.

It first identifies the leaf component, extracts its skeleton and identifies potential cut nodes using the distance transformation, and in the end selects the optimal cut nodes by a loss function. We applied this algorithm to leaves of the plant *C. hirsuta*, a model organism for dissected leaf development that moved into focus due to its leaf shape and its close relationship to *A. thaliana* [22].

As the processing of a single leaf image takes only a few seconds, our method is applicable in high-throughput applications where a large variety of measurements for thousands of leaves have to be taken. Based on the separation into the phenotypically interesting units we calculate several morphological measurements. These measurements include shape parameters of the leaflet, like leaflet area, eccentricity and perimeter. Additionally, our algorithm analyses the local position of a leaflet in the whole leaf, e.g. the distance of the leaflet from the rachis (petiolule length) or the distance of the leaflet from the terminal leaflet (inter-rachis, terminal rachis length). This is a complete representation of all morphological features of a dissected leaf, which are useful for a wide spectrum of applications—e.g for mapping the genetic basis of variation in individual or combined shape features, investigation of leaf shape plasticity in responses to environmental variables or clustering of mutant phenotypes.

To test the power of our algorithm we applied it to the two *C. hirsuta* accessions Nz from New Zealand and Ox originating from Oxford. We show that entire leaf features as well as features for individual leaflets are able to discriminate between the two accessions. Our open-source software is a versatile tool that enables QTL analysis of leaf morphological variation in large mapping populations.

## Methods

### Plant growth conditions

The two *C. hirsuta* accessions Ox and Nz were grown under long day conditions in the greenhouse. The leaf of the fifth leaf node was harvested at identical developmental state—at flowering when the inflorescence was 15 cm of height. It was digitized with the Epson V700 Photo scanner at 600 dpi.

### Image processing and feature detection

Image skeletonization and Gaussian mixture clustering were applied using the particular methods in the Matlab image processing and statistics toolbox. Shape features for the whole leaf and the leaflets were calculated using the standard Matlab methods. In order to calculate the petiolule length, two points at the start and the end of the petiolule were calculated. These points were defined by the cut of the line between opposite border points *b*1 and *b*2 and the skeleton. The petiolule was afterwards determined by counting the number of skeleton pixels (graph nodes) between these points. The rachis length was calculated by counting the number of skeleton pixels from the bottom point to the intersection between terminal leaf border points and the skeleton as explained above. The inter-rachis distance was calculated by searching in the graph the nearest branching node with the same orientation in direction of the terminal leaflet. Shape analysis was carried out by MorphoJ [25]. Comparison of different accessions was performed using discriminant factor analysis in MorphoJ. Manual measurements were determined using the software ImageJ [38].

### Performance and scalability

A set of 60 images was processed, consisting of 28 images of the Nz accession and 31 images of the Ox accession. The images had a resolution of 600 dpi, resulting in a image size of $4200\times 1200$ pixel. Processing of an average image took in total about 14 s on a MacBook Pro (1.4 GHz Intel Core i5, 4 GB RAM). The segmentation of the whole leaf component of a single image by Gaussian Mixture clustering took about 1.5 s. The identification of crossing points and cut points and the segmentation of single leaflets took about 2 s. Calculation of the features and generation of the output plots took the remaining time (about 10 s).

### Software and availability

All analysis steps were implemented in Matlab and are combined in the software MowJoe. This software tool provides a rudimental graphical user interface in which a folder with leaf images can be processed. The software tool, as well as the Matlab source code and raw and processed data were published according to [30] and can be found at https://github.com/Henrik86/Mow_Joe (10.5281/zenodo.1181810).

## Additional file

**Additional file 1: Fig. S1.** Comparison of different thresholding methods. **Fig. S2.** Manual measurements of rachis and petiolule. **Fig. S3.** Comparison of accessions by different statistics.
