# Supplementary material for: MowJoe: a method for automated-high throughput dissected leaf phenotyping
Source: Plant Methods. 2018 Mar 26;14:27. doi: 10.1186/s13007-018-0290-y (PMC5868070; doi:10.1186/s13007-018-0290-y)
Supplement: Supplementary file 1 — Additional file 1: Fig. S1. Comparison of different thresholding methods. Fig. S2. Manual measurements of rachis and petiolule. Fig. S3. Comparison of accessions by different statistics. [file 13007_2018_290_MOESM1_ESM.pdf]

MowJoe: A method for automated-high throughput compound leaf phenotyping

Henrik Failmezger[1,2], Janne Lempe[1], Nasim Khadem[2] , Maria Cartolano[1], Miltos Tsiantis[1], Achim Tresch[1,3]

[1]Max Planck Institute for Plant Breeding Research, Carl-von-Linné-Weg 10, 50829 Cologne, Germany, [2]Department of Biology, University of Cologne, Zülpicher Str. 47, 50674 Cologne, Germany, [3]Institute of Medical Statistics and Computational Biology, University of Cologne, Bachemer Strasse 86, 50931 Cologne, Germany

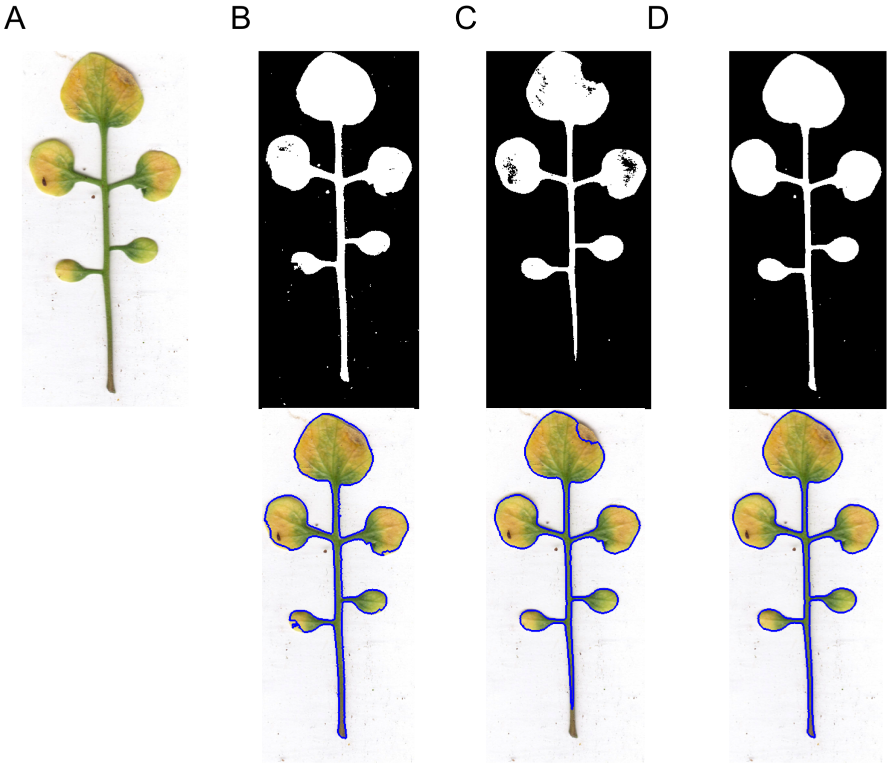

Fig. S1: Comparison of different thresholding methods. A: Original image. B: Otsu thresholding on the greyscale image. C: Otsu thresholding on the green component of the image. D: Gaussian mixture clustering of the SV space.

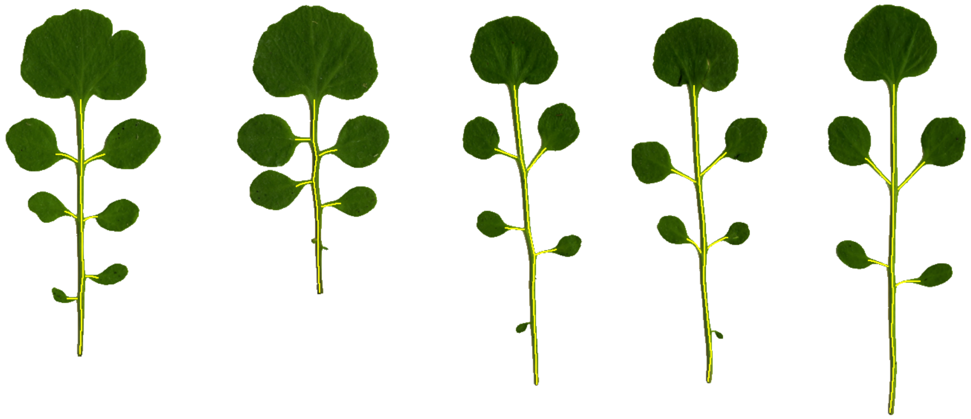

Fig. S2: Manual measurements of rachis and petiolule. The yellow lines were drawn by hand using ImageJ. The lengths of the line segments were calculated by ImageJ.

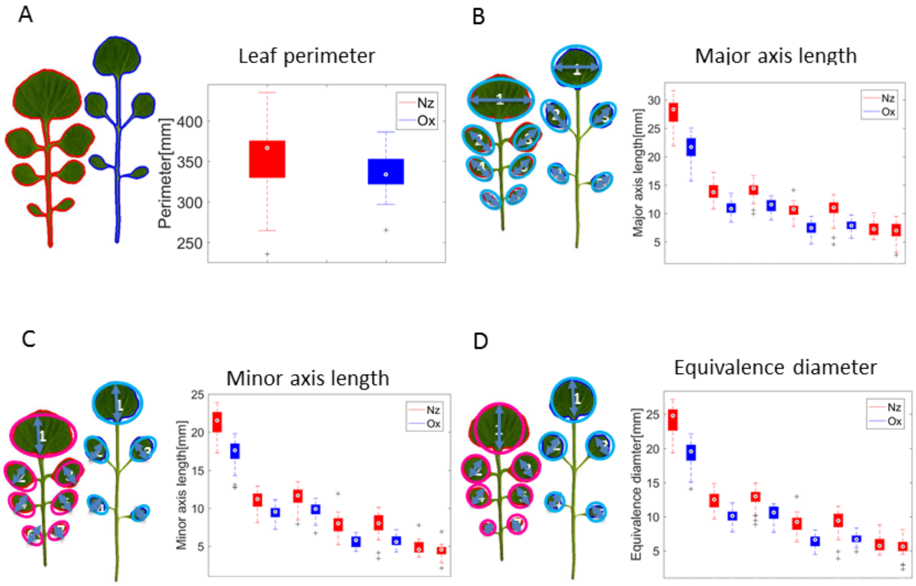

Fig. S3: Comparison of accessions by different statistics. Left leaf: Example of the New Zealand (Nz, red) accession, right leaf: Example of the Oxford (Ox, blue) accession. Leaflets are numbered according to their distance to the terminal leaflet. A: Perimeter of the whole leaf. B: Length of the leaflet's major axis. C: Length of the leaflet's minor axis. D: Diameter of the circle with the same area as the leafet.
